# Sensory modulation disorders in childhood epilepsy

**DOI:** 10.1186/s11689-015-9130-9

**Published:** 2015-10-23

**Authors:** Jolien S. van Campen, Floor E. Jansen, Nienke J. Kleinrensink, Marian Joëls, Kees PJ Braun, Hilgo Bruining

**Affiliations:** Department of Pediatric Neurology, Brain Center Rudolf Magnus, University Medical Center, Utrecht, The Netherlands; Department of Translational Neuroscience, Brain Center Rudolf Magnus, University Medical Center, Utrecht, The Netherlands; Department of Psychiatry, Brain Center Rudolf Magnus, University Medical Center, KC03.063.0, PO Box 85090, Utrecht, 3508 AB The Netherlands

**Keywords:** Seizures, Epilepsy, Sensory modulation, Excitation

## Abstract

**Background:**

Altered sensory sensitivity is generally linked to seizure-susceptibility in childhood epilepsy but may also be associated to the highly prevalent problems in behavioral adaptation. This association is further suggested by the frequent overlap of childhood epilepsy with autism spectrum disorder (ASD) and attention deficit hyperactivity disorder (ADHD), conditions in which altered behavioral responses to sensory stimuli have been firmly established. A continuum of sensory processing defects due to imbalanced neuronal inhibition and excitation across these disorders has been hypothesizedthat may lead to common symptoms of inadequate modulation of behavioral responses to sensory stimuli. Here, we investigated the prevalence of sensory modulation disorders among children with epilepsy and their relation with symptomatology of neurodevelopmental disorders.

**Methods:**

We used the Sensory Profile questionnaire to assess behavioral responses to sensory stimuli and categorize sensory modulation disorders in children with active epilepsy (aged 4–17 years). We related these outcomes to epilepsy characteristics and tested their association with comorbid symptoms of ASD (Social Responsiveness Scale) and ADHD (Strengths and Difficulties Questionnaire).

**Results:**

Sensory modulation disorders were reported in 49 % of the 158 children. Children with epilepsy reported increased behavioral responses associated with sensory “sensitivity,” “sensory avoidance,” and “poor registration” but not “sensory seeking.” Comorbidity of ASD and ADHD was associated with more severe sensory modulation problems, although 27 % of typically developing children with epilepsy also reported a sensory modulation disorder.

**Conclusions:**

Sensory modulation disorders are an under-recognized problem in children with epilepsy. The extent of the modulation difficulties indicates a substantial burden on daily functioning and may explain an important part of the behavioral distress associated with childhood epilepsy.

## Background

Epilepsy is among the most common chronic diseases in childhood, affecting 0.5–1 % of children [1]. Seizures cause a major disease burden and greatly affect cognitive and behavioral development [2]. Although seizures that are repetitively provoked by sensory stimuli (i.e., reflex seizures) only occur in a minority of patients [3–5], subclinically, children with epilepsy often exhibit altered neuronal responses to sensory stimulation compared with controls [6–14].

The capacity to regulate responses to sensory input in a graded and adaptive manner is generally referred to as sensory modulation [15]. Dunn proposed a model in which sensory modulation is characterized by four behavioral patterns: (1) sensory sensitivity—discomfort and distractibility caused by intense stimuli, (2) sensory avoiding—controlling or limiting the amount and type of sensations, (3) poor registration—lack of, or low awareness of sensations, and (4) sensory seeking—enjoyment of sensations and interest in increasing them [16]. Abnormal patterns of sensory modulation in children are known to interfere with effective learning, daily functioning [16], and interactions [17, 18], and are referred to as sensory modulation disorders (SMDs) [16–20]. SMDs are common in other neurodevelopmental disorders such as autism spectrum disorder (ASD) and attention deficit hyperactivity disorder (ADHD) [21–26], which are both frequently comorbid with epilepsy [27, 28]. Furthermore, SMDs have been linked to an imbalance between neuronal excitation and inhibition [29–33], which is a key feature of epilepsy, being present both during and in between seizures [34]. Taken together, we assumed that disordered sensory modulation might be a frequent problem in children with epilepsy which might contribute to behavioral distress and the behavioral problems observed in a significant number of children with epilepsy. As a first step to investigate the presence and role of sensory modulation in childhood epilepsy, we investigated (1) the prevalence of atypical behavioral responses to sensory stimuli and SMDs in children with epilepsy and (2) the association of these responses with comorbid symptoms of ASD and ADHD.

## Methods

### Patients

We retrospectively selected all children, aged 4–17 years, with active epilepsy (i.e., a definitive clinical diagnosis of epilepsy and seizures within 1 year prior to data collection), who consulted the pediatric neurology outpatient clinic of the University Medical Center Utrecht between September 2012 and October 2013. Children of whom caregivers could not recognize seizures, were well-controlled with AEDs (seizure-free for over a year) or since epilepsy surgery, and with non-Dutch-speaking caregivers were excluded. The study was approved by the institutional ethical committee. Questionnaires were sent to all caregivers. In case of initial non-response, caregivers were contacted by phone, asked for the reason of non-response, and motivated to complete the questionnaires. Parents or legal guardians and children aged ≥12 years provided informed consent. Children were retrospectively excluded when additional parental information revealed that children no longer fulfilled the inclusion criteria or had a developmental age <2 years (based on previous neurological and neuropsychological evaluation).

### Chart review

#### General demographics and epilepsy characteristics of responders and non-responders

Information on demographic and epilepsy characteristics, intellectual disability, and comorbidities (including ASD and ADHD) were extracted from patient files to enable comparison of responders and non-responders. Results of neuropsychological tests were evaluated for full-scale intelligence quotient (IQ) or developmental quotient (DQ, also known as mental developmental index), both referred to as “intelligence.” When neuropsychological test results could not be extracted from patient files, the original test reports were requested from parents. Epilepsy characteristics were classified according to the terminology proposed by the ILAE [35]. Epilepsy localization was classified as generalized or focal based on the diagnosis made by the treating pediatric neurologist. Focal epilepsies were further specified with respect to the hemisphere and lobe of origin. Characteristics of responders and non-responders were compared to estimate selection bias.

### Questionnaires

#### Additional information on general demographics and epilepsy characteristics of responders

Caregivers were questioned about the age of seizure onset, seizure frequency, current use of medication, comorbidities, psychiatric family history, and school type by questionnaire. They were asked to complete this questionnaire together with their child if possible. Anti-epileptic drugs used were classified according to their main known mechanism of action as γ-aminobutyric acid (GABA) enhancers, glutamate receptor antagonists, sodium channel blockers, calcium channel blockers, or others, according to Stafstrom (2010). In addition, information on reflex seizures and self-induction was obtained from patient files, and the response to photostimulation was extracted from EEG reports.

#### Sensory modulation

**Fig. 1:**
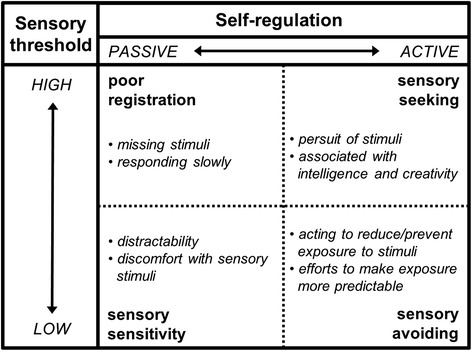
Sensory modulation quadrants: relationship between sensory threshold and self-regulation [22, 36, 37]. Figure adapted from [60]

Behavioral responses to sensory stimuli were quantified with the Sensory Profile method, consisting of well-validated questionnaires for different age groups [22, 36]. Behavioral responses to sensory stimuli are measured on four quadrants based on the interaction between the *sensory threshold* (i.e., the threshold for neurons to get activated by sensory stimuli, ranging from low to high) and the amount of *self-regulating behavior* (i.e., behavior used to regulate the sensory input, ranging from passive to active), see Fig. 1 [22, 36, 37]. Subscores also exist for sensory processing modalities, modulation and behavior, and emotional responses. Results correlate well with diagnoses of SMD made by occupational therapists and physiological responses to sensory stimuli [21, 23, 29, 31, 32, 38, 39].

For children aged 4–12 years, caregivers completed the parental Dutch version of the Sensory Profile (SP-NL) [22]. Children aged 13–17 years without intellectual disability completed the self-completed version of the Sensory Profile (Adolescent/Adult Sensory Profile [AASP]) [36]. For those with intellectual disability, this was done by caregivers. Norm-referenced scores were used for analysis. Z-scores were based on the standard deviations (SD) of the standardization samples published in the instruction manuals, available for SP-NL quadrants and subsections and for AASP quadrant self-scores [22, 36]. As results of SP-NL and AASP are indicated in the opposite direction, SP-NL z-scores were transposed so that higher scores indicated more symptoms. In this study, SMDs were defined as a score ≥2 SD above the mean on one or more of the quadrant scores [40]. To investigate the prevalence and nature of SMDs in children with epilepsy, quadrant scores (for all children) and subsections (for children 4–12 years) were compared with the reference group. In addition, associations between quadrant scores and demographic, epilepsy, and developmental characteristics were examined. To acquire more insight on the relation between sensory modulation and seizure precipitation, a question was added at the end of the subsection’s auditory, visual, vestibular, touch, and oral sensory processing (a non-validated tool), asking whether one or more of the items stated in that subsection could precipitate epileptic seizures.

#### Symptoms of ASD and ADHD

ASD and ADHD symptoms were evaluated by parental questionnaires well-validated for children of all ages. Previous studies have shown these disorders to go undetected in a large number of children with epilepsy [41]. Autism traits were measured using the Dutch Social Responsiveness Scale (SRS), a 65-item parental questionnaire [42]. Raw total scores were converted into age- and gender-specific T-scores using norms derived from the Dutch standardization sample, that were used for further analysis. ASD according to the SRS was defined as a score ≥75, according to the guideline [43]. ADHD symptoms were measured using the Dutch parental Strengths and Difficulties Questionnaire (SDQ), 25-item version [44, 45]. Scores on the hyperactivity-attention scale of this well-validated questionnaire were used as a measure of ADHD symptoms [46, 47]. ADHD according to the SDQ was defined as a score ≥7, according to the guideline [48, 49]. Typical development was defined as having no existing clinical diagnosis of ADHD or ASD, SRS score for ASD symptoms <75, SDQ score for ADHD symptoms <7, and (estimated) IQ ≥70.

### Data analysis

Group comparisons were performed with Fisher exact tests for binominal data, chi-square tests for non-dichotomous nominal data, *t* tests for continuous data, and Mann-Whitney tests for continuous data not meeting the assumptions of the *t* test. Unequal variance *t* tests were used to compare the study group with the combined reference group of the Sensory Profile (as a large difference in sample size exists and a larger variance is expected in the epilepsy group), followed by Bonferroni correction for multiple comparisons with adjustment for correlation between outcome parameters [50]. Correlations between continuous variables were tested with the Pearson correlation coefficient (*r*) or Spearman correlation coefficient (*ρ*) based on compliance to assumptions.

Associations between patient characteristics and Sensory Profile quadrant scores were examined with linear regression analyses. To simultaneously analyze outcomes on all four Sensory Profile quadrants, multivariate regression analyses were used. Patient characteristics with a univariable association of *p* < 0.1 were included as candidates into a multivariable regression model to maximize sensitivity. Variables were checked for multicollinearity using variance inflation factor (VIF) and tolerance, where a VIF ≥5 and tolerance ≤0.10 were considered to indicate a multicollinearity problem. Independent variables were removed by a backward stepwise selection procedure (threshold for removal *p* ≥ 0.05). To confirm robustness of the models, multivariable analyses were repeated using a forward selection procedure. To determine whether certain patient characteristics were related to scores on individual quadrants, post-hoc univariate linear regression analyses were performed per quadrant, including all characteristics with an association of *p* < 0.05 in the multivariate multivariable regression analysis as dependent variables and a single quadrant as dependent variable. All post-hoc tests were Bonferroni-corrected for multiple comparisons with adjustment for correlation between the quadrant scores [50].

Normality was visually inspected using Q-Q plots. Homogeneity of variance was evaluated with box plots. Results with a two-tailed *p* value of <0.05 were considered significant. Data were analyzed using SPSS, version 20.0.

## Results

### Patient selection

Questionnaires were sent to 375 patients. Sixty-six children were excluded retrospectively (exclusion criteria specified in Table 1). Of the remaining 309 children, 158 (51 %) responded. Compared with non-responders, responders consisted of less males (51 versus 65 %, this difference was most clear in the adolescent subgroup), and those with focal epilepsies showed more frontal and temporal seizure foci and less epilepsies with unknown localization. Median age of responders at completion of the questionnaires was 9.6 years. Seizure frequency in the past 3 months varied between 0 per 3 months and 15 per day (median two per week). Most children (94.7 %) were treated with anti-epileptic drugs at time of assessment. Demographics and epilepsy characteristics are shown in Table 2.

**Table 1:** Children excluded after receiving questionnaires

Exclusion criteria	Number of children excluded
Developmental age <2 years	41
Last seizure >12 months ago	9
Caregivers cannot recognize epileptic seizures	5
No understanding of Dutch language	5
Age at completion of questionnaires >17 years	3
Seizure freedom after epilepsy surgery	2
Death	1
Total	66

**Table 2:** Characteristics of responders and non-responders

Characteristics	Responders (*n* = 158)	Non-responders (*n* = 151)	*p* value
General			
Gender, % male	51	65	0.01
Age, median (range)	9.6 (4.1–16.7) yr	10.0 (4.1–16.6) yr	0.45
Epilepsy			
Seizure frequency (chart-based), median (range)	1/wk (0/3 mo–20/h)	1/wk (0/3 mo–12/h)	0.64
Seizure frequency (questionnaire-based), median (range)	2/wk (0/3 mo–15/day)	-	-
Etiology (chart-based), %			0.78
Genetic	11	13	
Structural	48	42	
Metabolic	4	5	
Unknown	36	39	
Seizure classification (chart-based), %			
Focal	44	45	0.91
Generalized	51	59	0.17
Epileptic spasms	2	1	0.62
Unknown	18	16	0.76
Localization (chart-based), distribution, %			0.06
Focal	57	48	
Multifocal	22	24	
Generalized	18	19	
Unknown	3	9	
Localization (chart-based), lobe, %			
Frontal	18	5	<0.001
Temporal	20	9	0.006
Parietal	15	8	0.16
Occipital	6	4	0.60
Unknown	15	25	0.07
Anti-epileptic treatment (questionnaire-based), %			
AED	98	-	-
GABA enhancers	50	-	
Glutamate receptor antagonists	8	-	
Sodium channel blockers	60	-	
Calcium channel blockers	25	-	
Other	32	-	
Vagal nerve stimulator	6	-	-
Ketogenic diet	6	-	-
Developmental			
Intelligence			
Intellectual disability (chart-based), %	42	47	0.36
IQ or DQ (test-based), median (range)	80 (23–148)	-	-
ADHD			
Diagnosis (chart-based), %	1	5	0.10
Symptoms (SDQ questionnaire-based), %			
Abnormal	38	-	-
Borderline	14	-	
Normal	48	-	
ASD			
Diagnosis (chart-based), %	12	11	0.86
Symptoms (SRS questionnaire-based), %			
Severe ASD symptoms	12	-	-
ASD symptoms	11	-	
Mild/moderate ASD symptoms	33	-	
No ASD symptoms	44	-	

*n* number of patients, *AED* anti-epileptic drugs, *intellectual disability* IQ or DQ <70, *IQ* intelligence quotient, *DQ* development quotient, *ASD* autism spectrum disorder, *SRS* Social Responsiveness Scale, *ADHD* attention deficit hyperactivity disorder, *SDQ* Strengths and Difficulties Questionnaire, *h* hour, *wk* week, *mo* month, *yr* year, *-* not applicable

### Sensory modulation

**Fig. 2:**
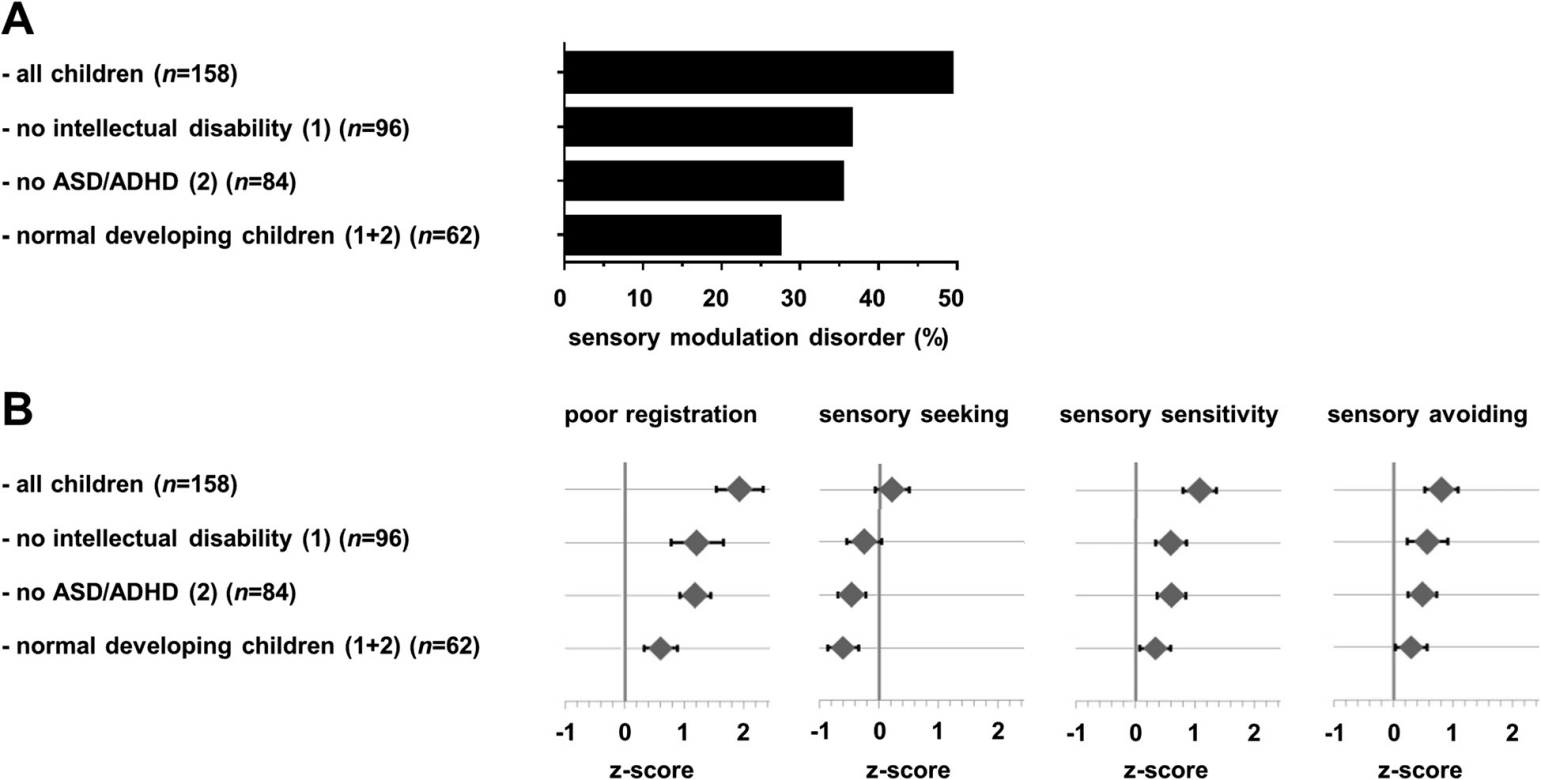
Sensory modulation in subgroups of children with epilepsy. **a** Percentage of sensory modulation disorders. **b** Sensory Profile quadrant scores. *n* number of patients, *ASD* autism spectrum disorder based on questionnaire, *ADHD* attention deficit hyperactivity disorder based on questionnaire. Values represent mean Z-scores and 95 % confidence interval, based on age-specific norms [22, 36]

**Fig. 3:**
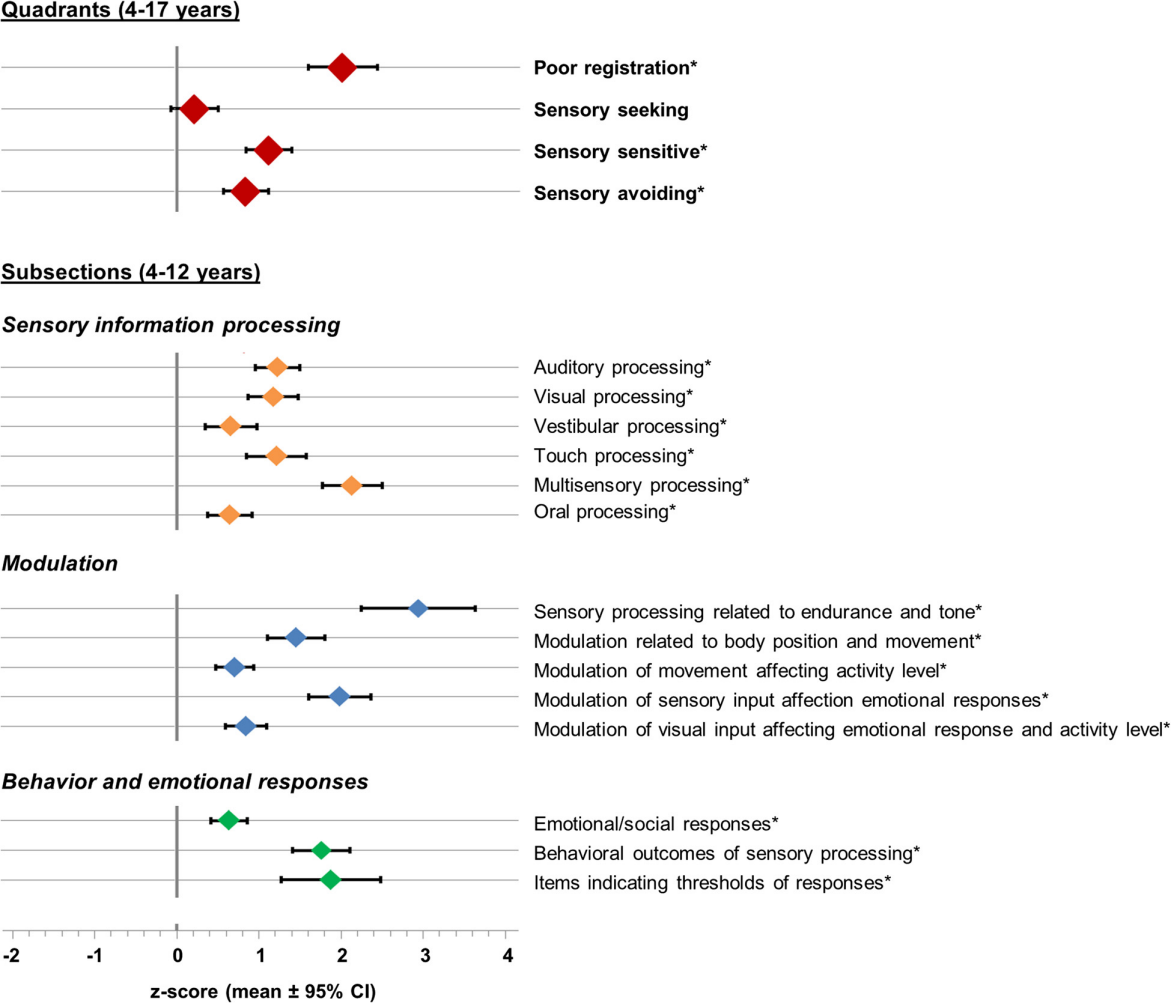
Sensory modulation scores in children with epilepsy. Sensory Profile quadrant scores of all children 4–17 years of age (*n* = 158) and subsection scores of children 4–12 years of age (*n* = 120). Values represent mean Z-scores (based on age-specific norms [22, 36]) and 95 % CI, *p value <0.001 compared with the reference population

SMDs were reported in nearly half (49 %) of the study cohort (Fig. 2). Although less frequent, SMDs were also reported in 27 % of the children with epilepsy who had a typical development. Compared with the reference group, mean Sensory Profile scores were significantly increased for all quadrants, except for sensory seeking (age 4–17 years), and for all subsections (age 4–12 years) (Fig. 3).

Quadrant scores were significantly correlated but did not meet criteria for multicollinearity. In univariable analyses, Sensory Profile quadrant scores were significantly associated with age, intelligence, age at seizure onset, epilepsy localization, number of AEDs, and ASD as well as ADHD symptom scores. No relation existed with etiology, epilepsy classification, or seizure frequency (Table 3). In multivariable analysis, age, intelligence, ASD symptoms, and ADHD symptoms remained significant. Post-hoc tests for the individual quadrants revealed that age was negatively correlated to all quadrants except “sensory sensitivity”; intelligence was negatively related to “sensory seeking”; ASD symptoms were positively correlated to all quadrants except “sensory seeking”; and ADHD symptoms were positively correlated to “sensory seeking” and “sensory sensitivity”.

**Table 3:** Relation between patient characteristics and sensory modulation

Characteristics	Analyses (F)		Post-hoc tests (beta)			
	Univar	Multivar^a^	Poor registration	Sensory seeking	Sensory sensitivity	Sensory avoiding
Gender, male	1.8	-	-	-	-	-
Age	24.8***	24.6***	−0.41***	−0.41***	−0.13	−0.46***
Intelligence (IQ or DQ)	14.2***	5.6***	−0.16	−0.21*	−0.12	0.15
Age at onset of epilepsy	8.7***	ns	-	-	-	-
Seizure frequency	2.0^	ns	-	-	-	-
Etiology						
Genetic	0.6	-	-	-	-	-
Structural	1.3	-	-	-	-	-
Metabolic	1.0	-	-	-	-	-
Unknown	ref	-	-	-	-	-
Distribution, hemisphere						
Left	3.9**	ns	-	-	-	-
Right	2.1*	ns	-	-	-	-
Both	ref	ref	-	-	-	-
Distribution, lobe						
Frontal	0.7	-	-	-	-	-
Temporal	1.5	-	-	-	-	-
Parietal	0.1	-	-	-	-	-
Occipital	1.3	-	-	-	-	-
Number of AEDs	4.6**	ns	-	-	-	-
AED-type						
GABA-enhancer	3.3*	ns	-	-	-	-
Glutamate antagonist	1.9^	ns	-	-	-	-
Sodium channel blocker	0.5	-	-	-	-	-
Calcium channel blocker	0.4	-	-	-	-	-
ASD symptoms (SRS)	13.9***	6.3***	0.29**	0.01	0.22*	0.45***
ADHD symptoms (SDQ)	18.3***	7.3***	0.10	0.33***	0.24**	−0.06

*Univar* multivariate univariable regression analysis, *multivar* multivariate multivariable regression analysis, *post-hoc tests* univariate multiple regression analyses per quadrant Bonferroni-corrected for multiple comparison, *IQ* intelligence quotient, *DQ* development quotient, *AED* anti-epileptic drug, *GABA* γ-aminobutyric acid, - not included in model, *ns* not significant

*p* values: ^<0.1 (only indicated for multivariate univariable regression analysis), *<0.05, **<0.01, ***<0.001

^a^Adjusted *R* ^2^ poor registration = 0.453, sensory seeking = 0.463, sensory sensitivity = 0.269, sensory avoiding = 0.340

### Sensory seizure precipitants

Items in subsections of the Sensory Profile were reported to precipitate epileptic seizures in 27 % of children. This was most often reported for items of auditory (16 %) and visual (12 %) processing, but also for vestibular (8 %), touch (6 %) and oral (5 %) processing items. With EEG, photosensitivity was demonstrated in four children (3 %), one of which had visual reflex seizures (with self-induction), and in the other three, a photoparoxysmal EEG response was described. Auditory reflex seizures were demonstrated in one child. Sensory Profile scores per subsection were positively correlated to the self-reported likelihood of these items to provoke epileptic seizures (*ρ* = 0.25–0.32, *p* = 0.001–0.006), except for oral processing (*ρ* = 0.04, *p* = 0.64).

## Discussion

This study shows that children with epilepsy report substantial problems in modulating their behavioral responses to sensory stimuli, generally referred to as SMDs. SMDs are known to perturb daily cognitive and behavioral functioning, which is confirmed by their correlation with ASD and ADHD morbidity in our sample. Interestingly, SMDs are also reported in 27 % of the typical developing children with epilepsy. Together, these findings indicate that SMDs are a substantial yet under-recognized problem in childhood epilepsy and might represent an important source of behavioral comorbidity.

Previous electrophysiological and imaging studies have shown increased sensitivity to sensory stimuli and overactive sensory processing networks in patients with epilepsy compared with controls [6–11]. Our findings suggest that these sensory processing defects may manifest as atypical *behavioral* responses to sensory stimuli in children with epilepsy and indirectly support the hypothesized role of the balance between excitation and inhibition in sensory modulation [33]. Consistently, behavioral symptoms relating to lower- and higher-order sensory processing abnormalities are frequently reported in ASD [51–53] and have been added to the diagnostic criteria for ASD in the Diagnostic and Statistical Manual of Mental Disorders [54]. In addition, sensory processing disorders are increasingly recognized as an independent clinical entity [55], which may relate to the phenotypic profile we observed in childhood epilepsy. Indeed, SMDs are also linked to other neurodevelopmental disorders such as ADHD [20–26]. Our observed association, between symptoms of these neurodevelopmental disorders and sensory modulation, further suggests that SMDs may explain an important part of the behavioral problems in childhood epilepsy.

Since epilepsy and seizures are related to neuronal *hyperexcitability*, we expected childhood epilepsy to be associated with *low sensory thresholds* and the experience of sensory overload. However, atypical sensory response patterns in our sample also included “poor registration,” generally attributed to a *high sensory threshold* [16]. A possible explanation might be found in compensatory inhibitory mechanisms [43], inducing a paradoxal *hypoexcitability*. The effect of AEDs on sensory modulation is likely to be minimal, as AED-type did not relate to sensory modulation patterns in a multivariable analysis, although the current study was underpowered to test effects of individual AEDs because of a large variety in (combinations of) AEDs that were used. Additionally, previous studies have shown differences in sensory processing of evoked potentials in patients with epilepsy irrespective of AEDs [6, 48, 49, 56]. It should also be noted that the children in our study had ongoing seizures despite polypharmacy, indicating that the neuronal excitation leading to seizures was probably still present. The high prevalence of SMDs in children with epilepsy on AED treatment, and the absence of a difference in prevalence between treatment groups, might suggest the prevalence to be even higher in a non-treated cohort. Another consideration is that comorbidity with ASD or ADHD is (partially) unrelated to the mechanisms causing seizures and therefore independently influences the pattern of SMDs. In children without comorbid ASD or ADHD, SMDs were also present, but they were overall less severe, which was particularly due to fewer behaviors related to “poor registration,” shifting the overall pattern of sensory modulation toward more sensory hypersensitivity (Fig. 3). Sensory processing scores for specific sensory modalities positively correlated to the reported likelihood of these modalities to precipitate seizures, providing additional evidence for a relation between disordered sensory modulation and neuronal hyperexcitability underlying childhood epilepsy. Altogether, although their relationship is not straightforward and different processes might interact, our findings indicate an important relation between epilepsy, excitability, and sensory behaviors.

### Study design and limitations

Due to data collection by questionnaires, a certain amount of recall and selection bias could not be prevented. To minimize recall bias, only patients with active epilepsy were included. To address selection bias, we compared characteristics of responders to those of non-responders. The decreased prevalence of males in responders, especially in the adolescent subgroup, is in line with the sex difference in survey-response behavior that was earlier reported in adults [57], and results in an even distribution of males and females in the study cohort. Epilepsy is a heterogeneous disease with respect to underlying pathology and localization of the epileptic network. As frontal lobe pathology is often accompanied by behavioral problems, the increased frequency of frontal epilepsies in responders might suggest an overestimated prevalence of behavioral disorders. However, effects of epilepsy localization on sensory modulation are probably limited, as regression analysis revealed no relation with the sensory modulation quadrant scores. Validated questionnaires were used whenever available. However, information on general and epilepsy characteristics (including seizure precipitants) was obtained by patient chart review and additional parental information provided on a non-validated questionnaire.

This study was performed in a tertiary referral center for children with epilepsy, where disease severity is expected to be higher than in general practice. Although we found no relation between atypical sensory processing and measures of disease severity in multivariable analysis, caution should nevertheless be taken when extrapolating the results to all children with epilepsy. The influence of age found in this cohort, with more atypical scores in younger children, might be explained by the developmental trends in some sensory modalities in the first years of life [24, 58], in combination with high prevalence of developmental delay, resulting in a younger developmental age compared to the calendar age in our study cohort.

As for all cross-sectional studies, no evidence is provided on causality of the reported associations. Although altered sensory modulation may affect seizure-susceptibility, the changes in brain function caused by recurrent seizure activity might also influence sensory modulation. Additionally, sensory modulation was quantified using questionnaires for behavioral responses. Although the outcomes of these questionnaires correlate well to SMD diagnoses and physiological responses to sensory stimuli [21, 23, 29, 31, 32, 38, 39], unraveling differential effects of specific sensory modulation profiles is difficult as scores on different quadrants are correlated, corresponding to the clinical observation that behaviors of different quadrants (e.g., sensory sensitivity and sensory seeking behavior) can be observed in the same child. The pathophysiological mechanisms underlying this observation remain for further study. Furthermore, it is unclear to which extent atypical sensory responses of children with epilepsy are caused by active seeking or avoidance of seizure triggers. Although scores on specific subsections of the Sensory Profile were significantly correlated to the likelihood of the sensory stimuli mentioned in these subsections to provoke seizures, the higher scores on the quadrants representing a passive behavioral response compared with the active ones, might suggest that the SMDs in children with epilepsy are less likely to be caused by active coping mechanisms.

### Implications

The reported changes in sensory thresholds might pose a large daily burden on children with epilepsy and their caregivers and play a causal role in (part of) the behavioral problems associated with epilepsy. Interventions that promote self-regulation have been shown to be helpful for patients with difficulties in sensory modulation in other neurodevelopmental disorders [59] and might also be beneficial for children with epilepsy. Further studies on SMDs in childhood epilepsy are needed to unravel the relation between sensory processing, neuronal excitability, and epileptic seizures.

## Conclusions

In conclusion, this study shows that atypical behavioral responses to sensory stimuli are highly frequent in childhood epilepsy. Increased attention for SMDs in children with epilepsy could benefit treatment and care.

## Abbreviations

ADHD: attention deficit hyperactivity disorder
AED: anti-epileptic drug
ASD: autism spectrum disorder
IQ: intelligence quotient
SD: standard deviation
SMD: sensory modulation disorder
